# Lymphocyte activation gene 3 is increased and affects cytokine production in rheumatoid arthritis

**DOI:** 10.1186/s13075-023-03073-z

**Published:** 2023-06-07

**Authors:** Janni Maria Pedersen, Aida Solhøj Hansen, Cæcilie Skejø, Kristian Juul-Madsen, Peter Junker, Kim Hørslev-Petersen, Merete Lund Hetland, Kristian Stengaard-Pedersen, Mikkel Østergaard, Bjarne Kuno Møller, Lene Dreyer, Ellen-Margrethe Hauge, Malene Hvid, Stinne Greisen, Bent Deleuran

**Affiliations:** 1 Department of Biomedicine, Aarhus University, C.F. Møllers Alle 6, 8000 Aarhus C, Denmark; 2 Department of Acute Medicine and Trauma Care, Aalborg University Hospital, Aalborg, Denmark; 3 Department of Rheumatology C, Odense University Hospital & Institute for Clinical Research, University of Southern Denmark, Odense, Denmark; 4 Danish Hospital for the Rheumatic Diseases, University of Southern Denmark, Odense, Denmark; 5 DANBIO and Copenhagen Centre for Arthritis Research (COPECARE), Centre for Rheumatology and Spine Diseases, Centre of Head and Orthopaedics, Rigshospitalet Glostrup, Glostrup, Denmark; 6 Department of Clinical Medicine, University of Copenhagen Faculty of Health and Medical Sciences, Copenhagen, Denmark; 7 Department of Rheumatology, Aarhus University Hospital, Aarhus, Denmark; 8 Department of Clinical Immunology, Aarhus University Hospital, Aarhus, Denmark; 9 Center for Rheumatic Research Aalborg, Department of Rheumatology, Aalborg University Hospital, Aalborg University, Aalborg, Denmark; 10 Department of Clinical Medicine, Aarhus University Hospital, Aarhus, Denmark

**Keywords:** Rheumatoid arthritis, LAG-3, Inflammation, Galectin-3, Co-inhibitory receptors

## Abstract

**Background:**

Lymphocyte activation gene-3 (LAG-3) inhibits T cell activation and interferes with the immune response by binding to MHC-II. As antigen presentation is central in rheumatoid arthritis (RA) pathogenesis, we studied aspects of LAG-3 as a serological marker and mediator in the pathogenesis of RA. Since Galectin-3 (Gal-3) is described as an additional binding partner for LAG-3, we also aimed to study the functional importance of this interaction.

**Methods:**

Plasma levels of soluble (s) LAG-3 were measured in early RA patients (eRA, *n* = 99) at baseline and after 12 months on a treat-to-target protocol, in self-reportedly healthy controls (HC, *n* = 32), and in paired plasma and synovial fluid (SF) from chronic RA patients (cRA, *n* = 38). Peripheral blood mononuclear cells (PBMCs) and synovial fluid mononuclear cells (SFMCs) were examined for LAG-3 expression by flow cytometry. The binding and functional outcomes of LAG-3 and Gal-3 interaction were assessed with surface plasmon resonance (SPR) and in cell cultures using rh-LAG3, an antagonistic LAG-3 antibody and a Gal-3 inhibitor.

**Results:**

Baseline sLAG-3 in the plasma was increased in eRA compared to HC and remained significantly elevated throughout 12 months of treatment. A high level of sLAG-3 at baseline was associated with the presence of IgM-RF and anti-CCP as well as radiographic progression. In cRA, sLAG-3 was significantly increased in SF compared with plasma, and LAG-3 was primarily expressed by activated T cells in SFMCs compared to PBMCs. Adding recombinant human LAG-3 to RA cell cultures resulted in decreased cytokine secretion, whereas blocking LAG-3 with an antagonistic antibody resulted in increased cytokine secretion. By SPR, we found a dose-dependent binding between LAG-3 and Gal-3. However, inhibiting Gal-3 in cultures did not further change cytokine production.

**Conclusions:**

sLAG-3 in the plasma and synovial fluid is increased in both early and chronic RA patients, particularly in the inflamed joint. High levels of sLAG-3 are associated with autoantibody seropositivity and radiographic progression in eRA, and LAG-3 plays a biologically active role in cRA by decreasing inflammatory cytokine production. This functional outcome is not affected by Gal-3 interference. Our results suggest that LAG-3 is a faceted regulator of inflammation in early and chronic RA.

**Supplementary Information:**

The online version contains supplementary material available at 10.1186/s13075-023-03073-z.

## Background

Rheumatoid arthritis (RA) is a chronic inflammatory joint disease characterized by progressive cartilage and bone destruction [1]. Activated CD4 + T cells are important conductors of nearly all aspects of the inflammation observed in RA, including the formation of osteoclasts leading to joint destruction [2]. CD4 + T cells engage with HLA class II molecules on antigen-presenting cells. A strong association between RA and MHC class II locus HLA-DRB1 is well recognized, supporting that MHC class II-driven antigen presentation is pivotal in RA pathogenesis and progression [3].

Co-inhibitory receptors (CIRs) such as cytotoxic T-lymphocyte-associated protein 4 (CTLA-4), programmed cell death protein 1 (PD-1), and lymphocyte activation gene 3 (LAG-3) play central roles in counteracting the pro-inflammatory processes by downregulating effector T cell activity [4, 5].

LAG-3 is a transmembranous protein of the immunoglobulin superfamily mainly identified in activated human T cells, regulatory T cells (Tregs), and NK cells [6, 7]. LAG-3 is highly homologous to CD4 and primarily recognizes stable peptide-MHC class II (pMHCII) complexes with higher affinity than CD4 [8, 9]. Cross-linking of LAG-3 with pMHCII leads to bidirectional inhibition. In the T cell, it reduces proliferation and IL-2 and IFNγ production [8]. In the antigen-presenting cell, CD86 expression and IL-12 production is reduced [9]. The mechanism of downstream signaling and consequent inhibitory function in both directions remain poorly defined [6, 10, 11]. LAG-3 thus plays an important immunomodulatory role in coordination with other CIRs, like PD-1, to maintain immunological self-tolerance [7, 12–14].

A naturally occurring soluble LAG-3 (sLAG-3) is shed from the cell surface by proteolytic cleavage mediated by metalloproteases ADAM10 and ADAM17 and is considered a surrogate marker for the amount of LAG-3 on the cell surface [8]. Soluble LAG-3 is a monomer and does not bind MHCII with any appreciable affinity [6]. In humans, high levels of sLAG-3 are associated with a less favorable disease outcome in cancer, and the addition of recombinant Fc-bound LAG-3 (Fc-LAG-3) has an anti-apoptotic effect in vitro [15, 16]. Besides effector T cells, regulatory T cells also express LAG-3; however, its role in different cell types remains unclear [17–19].

Apart from pMHCII, other LAG-3-binding proteins have been reported, including LSECtin, fibrinogen-like protein 1 (FGL-1), and galectin-3 (Gal-3) [20–22]. Gal-3 is abundant in RA plasma and joints and found to be a proinflammatory molecule involved in tissue destruction in RA [23, 24]. Gal-3 is unique among the galectin family members due to the presence of both a carbohydrate recognition domain and an oligomerization domain that enables Gal-3 to cross-link its binding targets and create large multimeric structures [25]. Gal-3 could potentially neutralize, or potentiate, both the membrane-bound and soluble form of LAG-3 according to LAG-3s expression of Gal-3 binding sites [5, 26].

Little is known about LAG-3 expression and its role in autoimmune diseases including RA. The anti-inflammatory capacity of LAG-3, the similarity with CD4, and the binding to pMHCII make it an intriguing target for investigation in RA. Here, we demonstrate that LAG-3 is elevated in patients with early and chronic RA and is associated with signs of more severe disease. Furthermore, we show that LAG-3 plays a biologically active role in suppressing proinflammatory cytokine secretion in RA, an effect that remains unchanged when inhibiting the LAG-3/Gal-3 interaction.

## Materials and methods

### Patients and samples

A set of plasma samples was obtained from patients enrolled in the Danish multicenter study OPtimized treatment in Early RA [OPERA; registered at ClinicalTrials.gov (NCT00660647)]. OPERA is a randomized, double-blind, placebo-controlled trial. Patients were adults, treatment-naïve, fulfilling the ACR 1987 revised criteria for RA, and with a disease duration of less than 6 months. The details of the study design have been published elsewhere [27]. Upon entry, patients were randomized to conventional methotrexate (MTX) treatment combined with an aggressive regimen of intra-articular betamethasone injections, with (*n* = 49) or without (*n* = 50) adalimumab (ADA) 40 mg SC every other week. For the current study, the two groups were merged as no difference between patients was observed in this analysis. Plasma samples (*n* = 99) and clinical data were collected at the initiation of treatment and after 6, 12, and 24 months. Samples were examined for sLAG3 at baseline and 12 months after treatment initiation. Radiographic measurements were obtained at inclusion in the study and 1 and 2 years after inclusion. Radiographic scoring used the Total Sharp Score (TSS) system which assesses joint space narrowing (JSN) and bone erosion (BE). In this study, we made use of the CRP-based Disease Activity Score using 4 variables (DAS28CRP) as a composite marker of disease activity, immunoglobulin M rheumatoid factor (IgM-RF), and anti-cyclic citrullinated peptide antibodies (anti-CCP), all obtained as a part of the OPERA study [27]. Clinical data were acquired at the same time points as the plasma samples were collected. Clinical and biochemical patient characteristics are presented in Table 1.

**Table 1:** Clinical characteristics and disease markers in 99 patients with early RA and 32 healthy controls

	**Early RA, baseline**	**Early RA, 12 months**	**Healthy controls**
**Gender, women (%)**	65 (66)		22 (67)
**Age (years)**	55 (46–64)		56 (51–64)
**DAS28CRP**	5.6 (4.8–6.5)	2.0 (1.8–2.8)	
**Swollen joint count (0–28)**	8 (5.0–13)	0 (0–0)	
**Tender joint count (0–28)**	11 (7.0–18)	0 (0–1.0)	
**Swollen joint count (0–40)**	11 (7.0–19)	0 (0–0)	
**Tender joint count (0–40)**	18 (11–25)	0 (0–2.3)	
**VAS doctor global (0–100 mm)**	53 (38–73)	2.0 (0–10)	
**CRP (mg/ml)**	15 (7.0–49)	7.0 (7.0–8.0)	
**CDAI (0.7–82)**	32 (23–44)	2.4 (0.28–5.4)	
**IgM-RF (% positive)**	67.7	–	
**Anti-CCP (% positive)**	58.6	–	
**TSS (% positive)**	20.0	81.8	
Δ**TSS**	–	1 (0–3)	

IgM-RF and anti-CCP were not measured at 12 months

Data are expressed as median with interquartile range (IQR)

*DAS28CRP* Disease activity score for 28 joints based on C-reactive protein, *CDAI* Clinical Disease Activity Index, *IgM-RF* IgM rheumatoid factor, *CCP* Cyclic citrullinated peptide, *TSS* Total Sharp Score

A cross-sectional sample set of plasma and synovial fluid (SF) was collected in EDTA tubes from patients with chronic (c) RA (*n* = 38, disease duration > 7 years) at the outpatient clinic at Aarhus University Hospital at the time of therapeutic arthrocentesis during a flare. Due to the cross-sectional design of this cohort, samples from earlier in their disease course were not available. The plasma and SF were isolated following centrifugation and kept at − 80 °C until analysis. Peripheral blood mononuclear cells (PBMCs) and synovial fluid mononuclear cells (SFMCs) were isolated using Ficoll-Pacque density gradient centrifugation and stored in freezing media (70% RPMI-1640, 20% fetal calf serum (FCS) and 10% dimethyl sulfoxide (DMSO)) at − 135 °C until the time of analysis.

Plasma samples from self-reportedly healthy age- and gender-matched donor controls (HC, *n* = 32) were obtained from the blood bank at Aarhus University Hospital.

### Quantification of protein

Quantification of sLAG3 in the plasma and SF was performed by a commercially available ELISA kit (Invitrogen, LAG-3 Human ELISA kit # BMS2211) according to the manufacturer’s instructions with the following exception: All samples were diluted 1:10 (plasma) or 1:20 (SF) in sample diluent supplemented with immunoglobulin in order to block for heterophilic antibodies [28]. All samples were analyzed in duplicate using the average of the optical density (OD) values to calculate concentrations. Values below the detection limit (6.25 pg/ml) were assigned the value of the detection limit. The ELISA was validated in accordance with previously published protocol showing no influence of rheumatoid factors or heterophilic antibodies [28]. Multiplexed protein expression in cell supernatants were measured by Meso Scale Discovery (V-PLEX Proinflammatory Panel 1 Human Kit) according to the manufacturer’s guidelines (Meso Scale Diagnostics).

### Cell culture

As an ex vivo model for RA, human PBMCs and SFMCs were cultured in RPMI supplemented with 10%FCS, 1% HEPES, 1% GlutaMAX, 1% penicillin/streptavidin 15 µg/ml, and gentamycin at a concentration of 1 × 10^6^ cells/ml, for 48 h, as previously described (*n* = 10) [29]. For the stimulation experiments, recombinant human LAG-3 (0.5 μg/ml, R&D Systems, Cat.2319-L3-050), neutralizing anti-LAG-3 antibody (10 μg/ml, 17B4, BPS Bioscience, Cat.71219), and/or Galectin-3 inhibitor (10 μM, GB0149, Galecto) were used. Pre-stimulation with plate-bound CD3 (0.5 μg/ml, OKT3, R&D Systems) and soluble CD28 (0.5 μg/ml, BD Bioscience) was conducted when applicable. Cells were cultured for 48 h at 37 °C without change of medium, and non-treated cultures or matching isotypes were used as controls. Supernatants were collected and kept at − 80 °C until cytokine measurements.

### Flow cytometry

PBMCs and SFMCs from chronic RA patients (*n* = 10) were stained for flow cytometry with the following antibodies: anti-CD4 PE-Cy7 (clone SK3, Cat. 557852), anti-HLA-DR PE (clone L243, Cat. 347401) (both from BD), anti-CD3 FITC (clone SK7, Cat.344804), anti-CD19 APC (clone HIB19, Cat. 302212), anti-CD45RO BV605 (clone UCHL1, Cat. 304238), and anti-LAG-3 BV421 (clone 11C3C65, Cat. 369314) (all from BioLegend). For the exclusion of dead cells, the samples were stained with LIVE/DEAD fixable near-IR (Molecular probe, Cat.L10119, Invitrogen). All samples were fixed and analyzed within 24 h using a Novocyte Flow cytometer (Beckmann Coulter), and data were processed in the FlowJo 8.8.3 software (Tree Star Inc., USA). For the analysis of LAG-3 expression on activated T cells, 0.5 mil PBMCs or SFMCs were activated with Dynabeads human T activator CD3/CD28 antibodies (Gibco, Cat. 11131D, Thermo Fisher) in a bead-to-cell ratio of 1:2 for 48 h at 37 °C and 5% CO_2_. For the last 4 h of the incubation period, the cells were treated with 20 µg/ml Brefeldin A (Sigma-Aldrich, Cat.B7651). The cells were stained for flow cytometry using the following antibodies: anti-CD3-FITC (clone UCHT-1, Cat.300402, BioLegend), anti-CD4-Brilliant Violet 605 (clone RPA-T4, Cat. 562658, BD Horizon), anti-LAG-3-Brilliant Violet 421 (clone 11C3C65, Cat. 369314, BioLegend), anti-PD-1-PE (clone EH12.2H7, Cat. 329906, BioLegend), and anti-IL-2-PE-Cy7 (clone MQ1-17H12, Cat.554566, BD). Prior to staining for the intracellular IL-2, the cells were fixed and permeabilized using the Transcription Factor Buffer Set (Cat.562574, BD Pharmingen) according to the manufacturer’s instructions. In all panels, antibodies were titrated to the optimal working concentration. Gating of positive and negative cells was achieved using fluorescence minus-one controls (FMO) and blanks (Fig. S1).

### Surface plasmon resonance (SPR)

The macromolecular interaction between LAG-3 and potential ligands was examined using a Biacore SPR reader. In short, either Fc:LAG3 or LAG-3 mAb was immobilized on the chip and the flow contained either Fc:LAG3, LAG-3 mAb, rh-Gal-3, or preincubated LAG3/Gal-3 with increasing concentrations. Determination of binding constants: The two-dimensional fits were made on the MATLAB 2012a platform (MathWorks) using the fitting tool EVILFIT version 3 software by Peter Schuck [30, 31]. In brief, input values matched the start and end injection time and included concentrations spanning from 0 to 6400 nM Gal-3 with a fixed concentration of Fc-LAG3 at 25 nm. The association phase was fitted from *t* = “injection start” plus 1 s to *t* = “injection end” minus 5 s. The dissociation phase was fitted from *t* = “injection end” plus 30 s to *t* = “injection end” plus 190 s.

The operator set boundaries for the distributions were uniformly set to limit *K*_*d*_ values in the interval from 10^−12^ to 10^−3^ M, and *k*_off_ values in the interval from 10^−6^ to 10^1^ s^−1^ to ensure comparable and best quality fits reflected in a high signal-to-rmsd ratio.

The distribution P (*k*_*a*_, *K*_*A*_) is calculated using the discretization of the equation:

$$R_{\mathrm{total}}=\int_{K_{A\min}}^{K_{A\max}}\int_{k_{a\min}}^{k_{a\max}}R\left(k_a,K_A,C_{\mathrm{analyte}},t\right)P\left(k_a,K_A\right)dk_adK_A$$

in a logarithmic grid of (*k*_*a*,*i*_, *K*_*A*,*i*_) values with 9 times 9 grid points distributed on each axis, respectively. This was done through a global fit to the association and dissociation traces at the above-mentioned analyte concentrations. Tikhonov regularization was used as described by Zhao et al. at a confidence level of *P* = 0.95 to determine the most parsimonious distribution that is consistent with the data, showing only features that are essential to fit the data [32].

## Statistical analysis

Statistical analyses and graphs were performed using GraphPad Prism 7.0 for Mac (GraphPad Software, La Jolla, CA, USA). Paired samples were compared by Wilcoxon’s signed rank test whereas non-paired data were analyzed using the Mann–Whitney *U*-test. The correlation was tested using Spearman’s rho (*ρ*). Parametric data results are specified as means with standard deviations whereas non-parametric data are indicated as medians with IQR. In all tests, the level of statistical significance was a two-sided *p*-value of less than 0.05.

## Results

### Plasma levels of sLAG-3 are elevated in early RA and associated with the presence of autoantibodies and the development of bone erosions

**Fig. 1:**
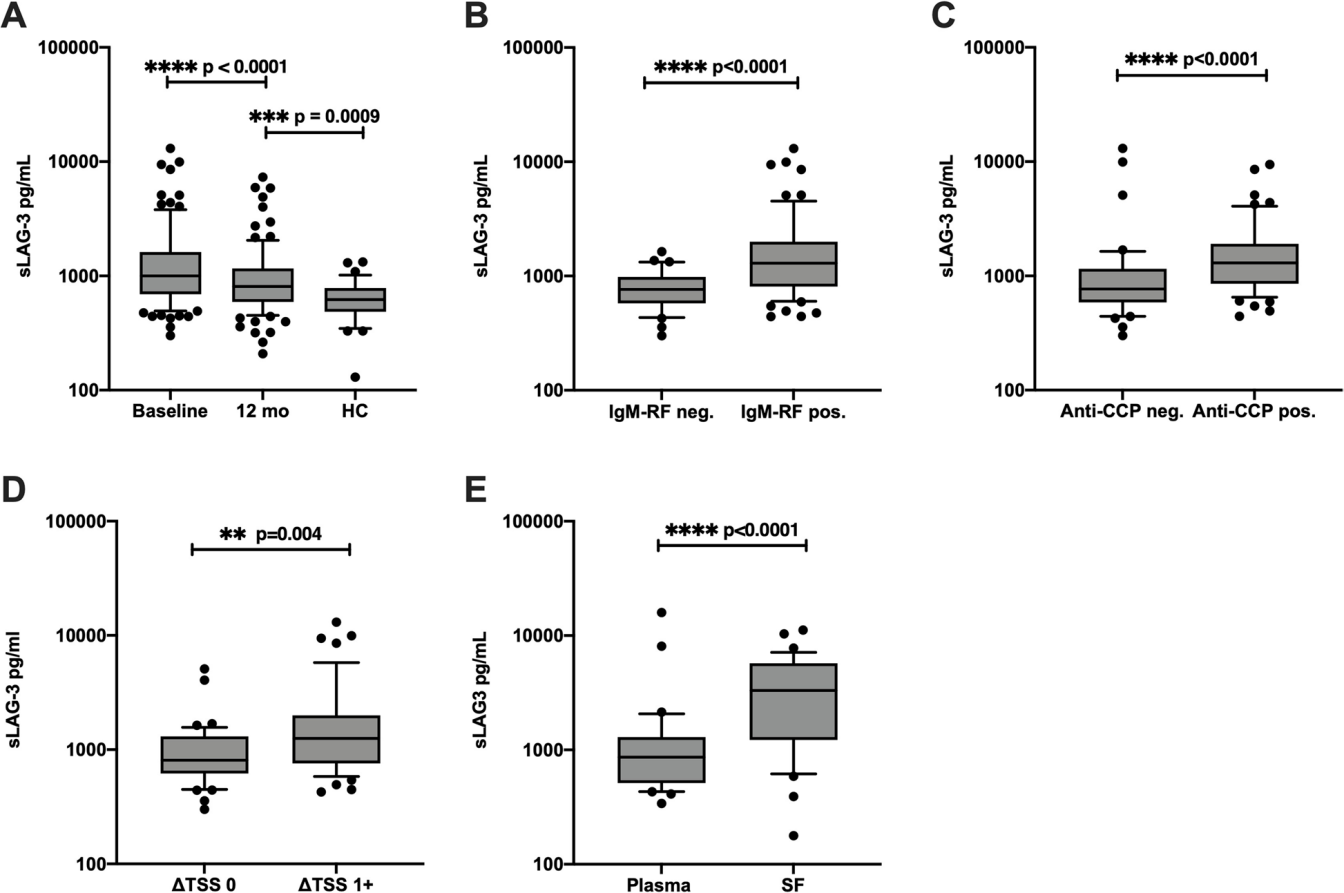
Levels of sLAG-3 in RA and HC. **A** sLAG-3 was measured in eRA (*n* = 99) at baseline and after 12 months of therapy and compared to sLAG-3 levels in healthy controls (*n* = 32). **B**, **C** At baseline, eRA patients (*n* = 99) were examined for levels of plasma sLAG-3, which were elevated in the IgM-RF-positive (*n* = 67) and anti-CCP-positive (*n* = 58) patients compared with seronegative patients. **D** Comparison of baseline sLAG-3 levels in eRA patients (*n* = 90) with disease progression measured by increased Total Sharp Score (ΔTSS 1 +) vs. patients without disease progression (ΔTSS 0) over a 2-year period. **E** Comparison of sLAG-3 in the plasma vs. synovial fluid in cRA (*n* = 38). Boxes indicate median and IQR, whiskers 10th–90th percentiles. Differences were analyzed using the Wilcoxon test or Mann–Whitney test to compare the two groups. The level of significance is indicated on the graph. IgM-RF, IgM rheumatoid factor; CCP, cyclic citrullinated peptide; TSS, Total Sharp score

To investigate the role of LAG-3 in RA pathogenesis, we first evaluated levels of sLAG-3 in an early RA cohort with specified clinical, biochemical, and radiological outcomes. In eRA patients, plasma levels of sLAG-3 were significantly increased at baseline (1000 pg/ml (691–1621 pg/ml), median (IQR)) compared to 12 months of treatment (809 pg/ml (592–1163 pg/ml) (*p* = 0.0001) and with HC (620 pg/ml (486–783 pg/ml), *p* < 0.0001) (Fig. 1A). Although sLAG-3 decreased during the 12 months’ treatment period (809 pg/ml (592–1163 pg/ml)), it remained significantly elevated compared with HC (636 pg/ml (511–914 pg/ml), *p* = 0.0009, Fig. 1A). Furthermore, we observed a linear correlation between sLAG-3 levels at baseline and at 12 months (*r* = 0.7, *p* < 0.0001) (Fig. S2A).

We then examined the association of sLAG-3 with baseline characteristics and disease severity. At baseline, eRA patients with positive IgM-RF or anti-CCP antibody titers had significant higher sLAG-3 plasma levels (1296 pg/ml (808–1998 pg/ml) and 1300 pg/ml (853–1909 pg/ml), respectively) than seronegative patients (763 pg/ml (578–981 pg/ml) and 768 pg/ml (587–1155 pg/ml), respectively; both (*p* < 0.0001), (Fig. 1B, C). We found no correlation of sLAG-3 with DAS28CRP as a marker of disease activity neither with age, gender, nor smoking. Linear regression analysis did not show a correlation between sLAG3 at baseline and the TSS score at baseline, but we observed that plasma levels of sLAG-3 at baseline were significantly higher in patients with radiographic progression (ΔTSS) after 12 months. This became increasingly significant after 24 months (Fig. 1D). Moreover, changes in sLAG-3 from 0 to 12 months were also positively correlated with progression in TSS from baseline to 24 months (ΔTSS) (*r* = 0.25, *p* = 0.005) (data not shown). To further examine the association between sLAG3 levels and the progression of TSS, we made a ROC curve to evaluate LAG-3’s strength as a predictive marker at the time of diagnosis. When splitting sLAG3 plasma levels in the two groups “non-erosive” versus “erosive” patients, sLAG3 shows an overall performance of 67% (AUC = 67%; *p* = 0.004) in association with progression on TSS (Fig. S2B). This again confirms that sLAG3 is associated with the development of erosions in eRA.

In early RA patients, sLAG-3 plasma levels remained elevated above the control level although exhibiting a temporal decline following treatment initiation. Moreover, sLAG-3 was correlated with the presence of autoantibodies, and sLAG-3 levels were also predictive for a subgroup of patients with a more severe disease course as high sLAG-3 levels were associated with increased radiographic progression.

### In chronic RA patients, LAG-3 is primarily expressed by synovial T cells in conjunction with PD-1

The joint is the major site of inflammation in RA. Therefore, we measured levels of sLAG-3 and the cellular expression of LAG-3 in paired samples of plasma and synovial fluid (SF) from chronic RA patients. Soluble LAG-3 was detectable in high amounts in SF in all of the patients. Significantly elevated levels of sLAG-3 were observed in the synovial fluid (3314 pg/ml (1225–5724 pg/ml)) compared with plasma (863 pg/ml (512–1296 pg/ml), *p* < 0.0001) (Fig. 1E). The level of sLAG-3 in the plasma from cRA patients did not significantly differ from eRA patients, neither at baseline (*p* = 0.09) nor at 12 months (*p* = 0.87).

**Fig. 2:**
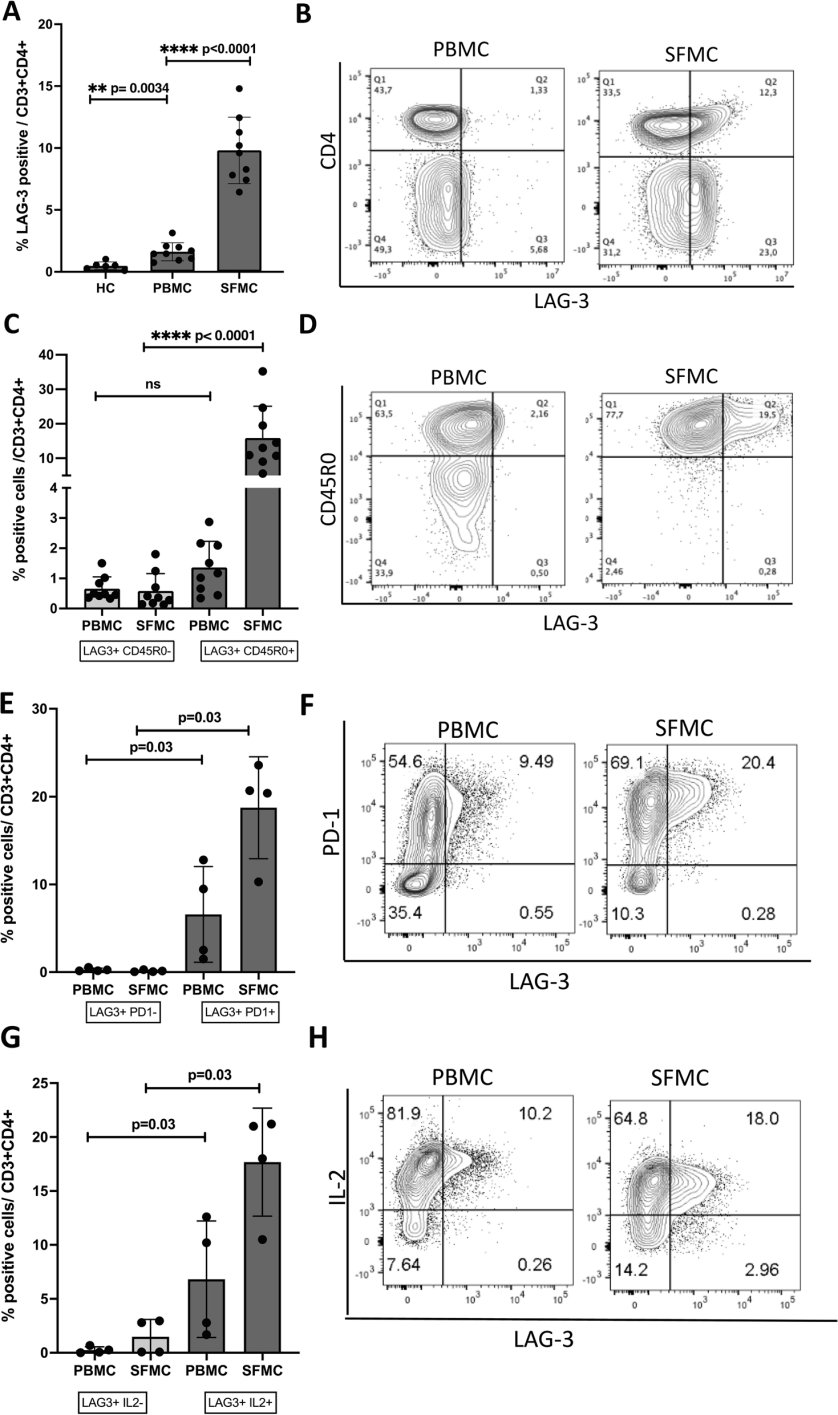
Cellular expression of LAG-3 in PBMCs and SFMCs. **A** Cellular expression of LAG-3 on CD3 + CD4 + T cells from PBMCs and SFMCs in HC (*n* = 6) and cRA (*n* = 9). **B** Representative FACS data from one of the cRA patients shown in the flow plot. **C** Distribution of LAG-3 + cells in relation to CD45R0 presented in the bar graph (*n* = 12) and representative FACS data from one of the cRA patients shown in the flow plot (**D**). **E** Percentages of LAG-3 + cells determined by PD-1 co-expression are presented in the bar graph (*n* = 4) and representative FACS data from one of the cRA patients are shown in the flow plot (**F**). **G** Percentages of LAG-3 + cells capable of IL-2 production or not (*n* = 4) with a representative flow plot in **H**. Bars indicate the median and whiskers standard deviation. The level of significance is indicated on the graph. Differences were analyzed using the paired *t* tests or Mann–Whitney test to compare the two groups

Next, we examined the cellular expression of LAG-3 in PBMCs and SFMCs from chronic RA patients and found an increased frequency of LAG-3 + T cells in SFMCs compared to PBMCs. This was the case for both CD4 + (9.6% vs. 1.5%; *p* < 0.0001; Fig. 2A) and CD4 − (12.3% vs. 1.8%; *p* = 0.003; Fig. S3) cells. In CD4 + T cells from cRA PBMCs, LAG3 expression was also more frequent than in healthy controls (1.5% vs. 0.5%; *p* = 0.0034). LAG-3 in both joint and peripheral blood was expressed primarily by CD45R0 + T cells, indicating that they are or previously have been activated. In CD4 + T cells, 13.0% were LAG3 + CD45R0 + vs. 0.36% LAG3 + CD45R0 − (Fig. 2C), whereas in CD4 − T cells, 23.9% were LAG3 + CD45R0 + vs. 2.9% LAG3 + CD45R0 − (Fig. S3B). We also examined CD19 + B cells for the expression of LAG-3 and found a small fraction of positive cells in both PBMCs and SFMCs but did not observe any distinctive patterns between individual subpopulations (Fig. S4).

The immunomodulatory function of LAG-3 is perceived to be coordinated with other CIRs including PD-1 [13, 33, 34]. Therefore, we next examined the cellular co-expression of LAG-3 and PD-1 by PBMCs and SFMCs in chronic RA patients and their capability to produce IL-2 after CD3/CD28 stimulation. We observed that all LAG-3 + T cells in both PBMCs (6.0% LAG3 + PD1 + vs. 0.2% LAG3 + PD1 −) and SFMCs (20.6% LAG3 + PD1 + vs. 0.01% LAG3 + PD1 −) were positive for PD-1 (Fig. 2E) and that nearly all LAG-3 + T cells expressed IL-2 (Fig. 2G). In PBMCs, 6.5% T cells were LAG3 + IL-2 + vs. 0.2% LAG3 + IL-2 − , and only a minor fraction of LAG-3-positive cells from SFMCs were IL-2 negative (19.5% LAG3 + IL2 + vs. 1.4% LAG3 + IL-2 −). Altogether, these data confirm that the inflamed joint during a flare is a major site of accumulation of LAG-3 expressing T cells and support that LAG-3 reflects T cell activation.

### Addition of rhLAG-3 decreases cytokine production in PBMC and SFMC cell cultures

**Fig. 3:**
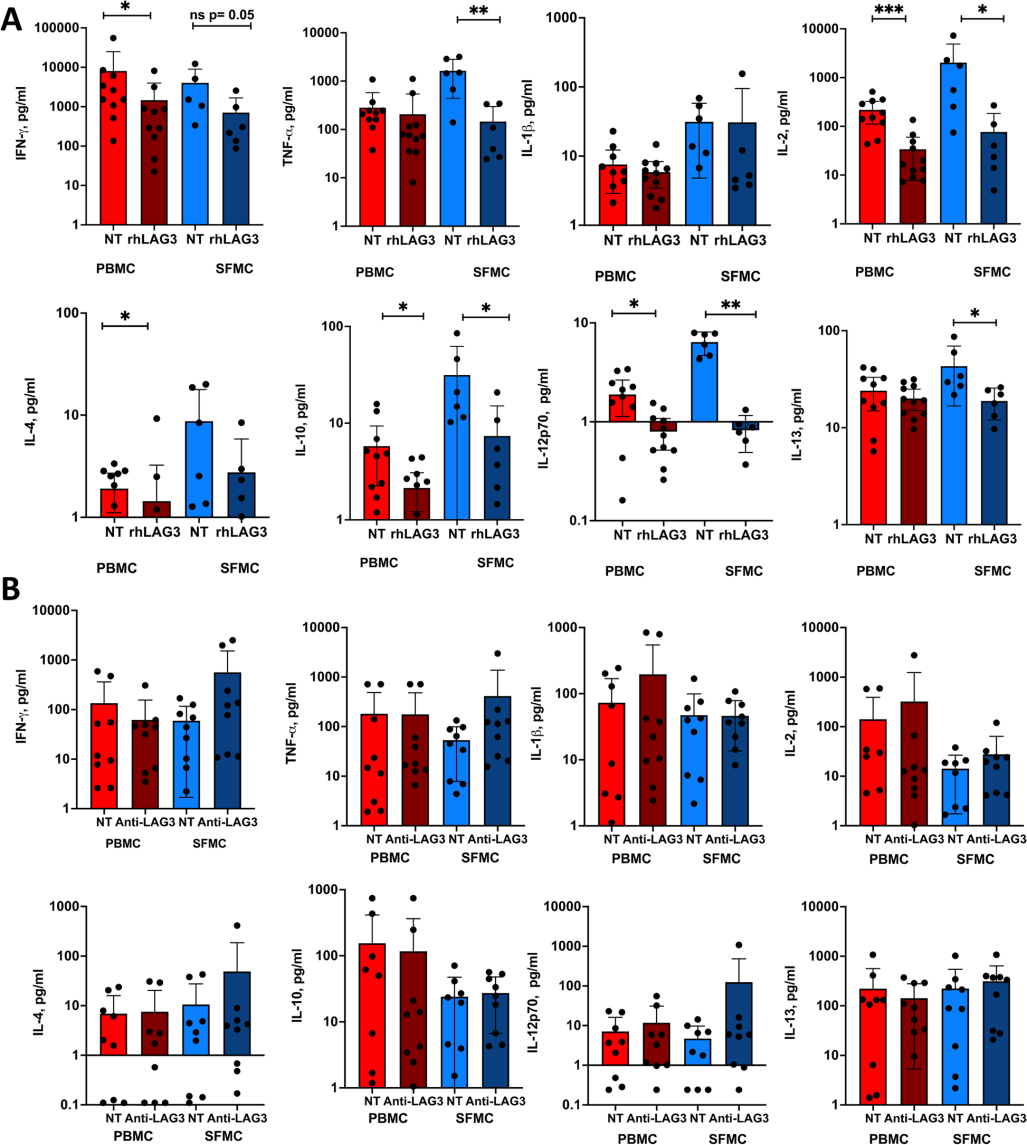
Effects of rhLAG-3 and antagonistic LAG-3 mAb on cytokine production. Production of cytokines in PBMC and paired SFMC cultures from cRA patients (*n* = 10) with or without the addition of recombinant human LAG-3 (**A**) or antagonistic LAG-3 mAb (**B**). The level of significance is indicated by * < 0.05. Differences were analyzed using the Mann–Whitney test to compare the two groups

After observing increased sLAG-3 and LAG-3 expression in the inflamed RA joint, we progressed to test the functional effect of adding either rhLAG-3 or antagonistic LAG-3 mAb in PBMC and SFMC cultures and evaluated cytokine production. Adding rhLAG-3 resulted in decreased synthesis of all examined cytokines after 48 h in both PBMCs and SFMCs, although not significant for IL-1β and IL-6 (Fig. 3A). In general, the decrease was more pronounced in SFMCs than PBMCs. Adding antagonistic LAG-3 mAb did not statistically significantly increase the cytokine production separately although an overall difference in “not-treated” and “treated” patients was found by ANOVA (*p* = 0.03) in SFMCs (Fig. 3B). This supports that LAG-3 plays a biological active role in RA particularly in the inflamed joint.

### Binding to Galectin-3 affects the interaction between LAG-3 and the antagonistic LAG-3 mAb

**Fig. 4:**
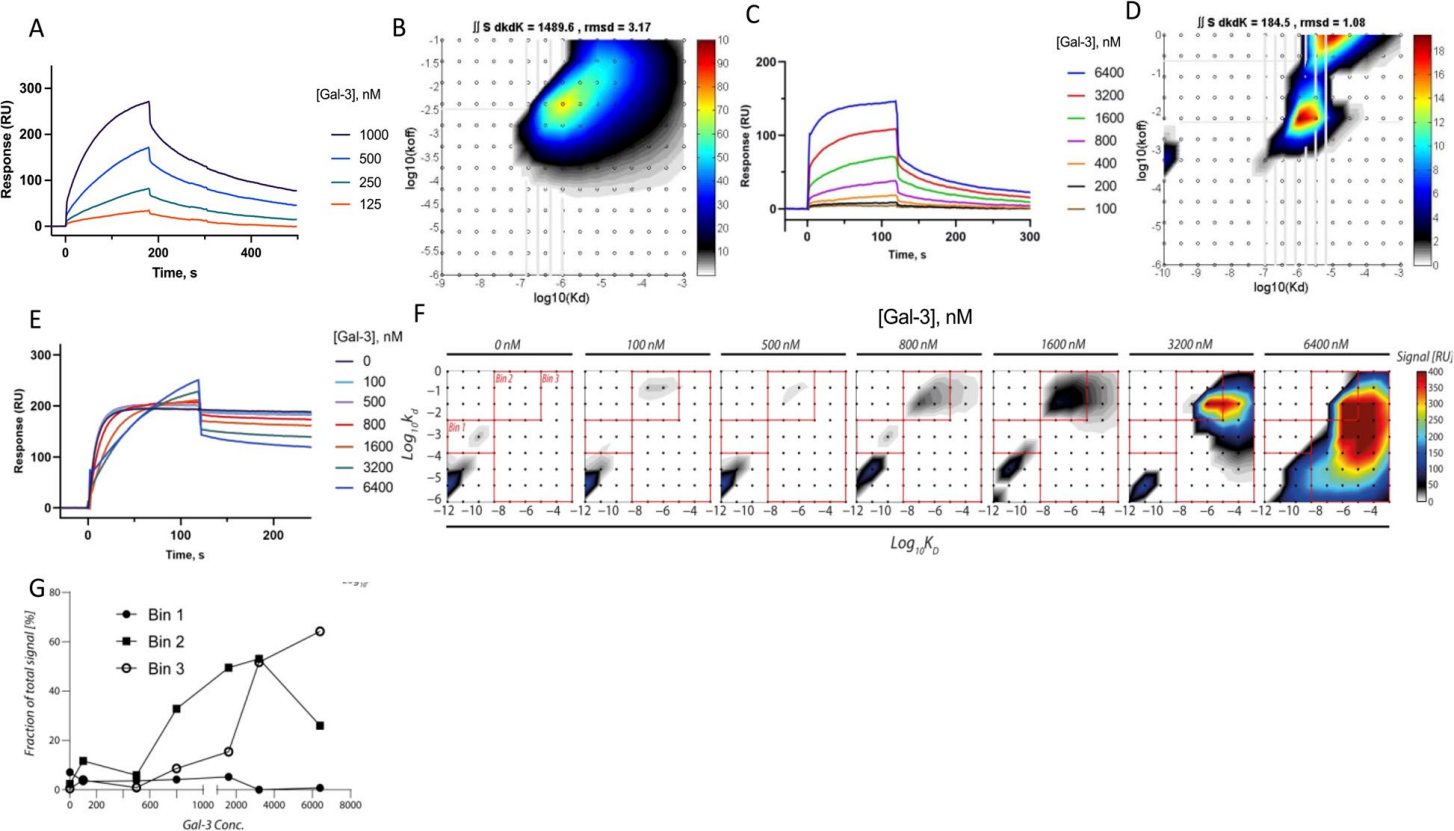
Surface plasmon resonance (SPR) results showing LAG-3, anti-LAG3 mAb, and Gal-3 binding kinetics. **A** SPR plot showing Gal-3 binding to LAG-3 in a dose-dependent way. **B** Fits to the experimental data were made with EVILFIT for the LAG-3/Gal-3 interaction. The distribution in ligand binding kinetics is shown in the two-dimensional grids. Each grid point represents a 1:1 interaction typified by *K*_*D*_ and *k*_*d*_. A third coordinate, indicated with a color scale, represents the abundance of these interactions.** C** SPR plot and corresponding EVILFIT curve (**D**) showing Gal-3 binding to the antagonistic LAG-3 Ab (17B4) without the presence of LAG-3. Kinetics in a similar range as Gal-3/ LAG-3 interaction is seen. **E** SPR plot showing how Gal-3 interferes with the LAG-3/antagonistic LAG3 antibody binding in a dose-dependent way. Chip with immobilized antagonistic LAG-3-Ab (17B4). Flow containing a constant Fc:LAG3 amount and increasing concentrations of human recombinant Gal-3 were added. **F** The interaction between LAG-3 and LAG-3 mAb as a function of Gal-3 concentration is plotted with two-dimensional fits. To quantify the development in the distribution of binding kinetics, three bins (Bin 1, Bin 2, and Bin 3) were defined, guided by the major types of interactions observed for LAG-3, Gal-3, and LAG-3 mAb. **G** From bin 1, we see a typical signal from a mAb interaction (*K*_*D*_ at 1 nM) at the lowest concentrations of Gal-3. This signal decreases with increasing Gal-3 concentrations and is lost above 1600 nM Gal-3. Bin 2 shows an intermediate state with complexes between LAG-3, Gal-3, and mAb that occurs with Gal-3 concentrations between 100 and 3200 nM Gal-3 (*K*_*D*_ 10 µM). Bin 3 shows direct binding between Gal-3 and anti-LAG-3-mAb (*K*_*D*_ 1 mM–1 µM)

Since the change in cytokine production was less clear when adding a neutralizing LAG-3 mAb, we aimed to determine if the outcome was affected by other binding partners to LAG-3. In addition to MHC-II, Gal-3 have been reported to bind LAG-3. We, and others, have shown that the levels of Gal-3 are elevated in RA [23, 24], and we therefore used surface plasmon resonance to study the interaction of rhLAG-3 and LAG-3 mAb with Gal-3. rhLAG-3 did bind Gal-3 in a dose-dependent manner, and saturation was not achieved at 1000 nM (Fig. 4A). In order to visualize the binding kinetics between rhLAG-3 and Gal-3, we performed EVILFIT to get at the 2D distribution of binding contributions. The *K*_*D*_ distribution spanned from 1 mM to 1 µM reflecting multiple bindings and/or multimerization (Fig. 4B). Moreover, we tested for Gal-3 interaction directly with the anti-LAG-3 mAb and confirmed this binding showing kinetics in a similar range as Gal-3 to rhLAG-3 (Fig. 4C, D).

We proceeded to test whether Gal-3 interfered with the interaction between LAG-3 and the antagonistic anti-LAG-3 mAb using surface plasmon resonance. Here, we immobilized the antagonistic LAG-3-mAb on the chip and examined the increasing concentrations of either rhLAG3 alone or complexes of rhLAG-3/Gal-3. We observed a clear dose-dependent inhibition of the LAG-3/anti-LAG3-mAb binding by increasing Gal-3 concentration, although saturation was not accomplished even with the addition of 6400 nM of Gal-3 (Fig. 4E). We once again performed EVILFIT to analyze the change in binding kinetics between LAG-3 and anti-LAG-3-mAb as a result of increasing Gal-3 concentrations. This visualization shows a typical signal from an Ag-mAb interaction (*K*_*D*_ at 1 nM) at the lowest concentrations of Gal-3 to an intermediate state with complexes between LAG-3, Gal-3, and mAb occurs with Gal-3 concentrations between 100 and 3200 nM Gal-3 (*K*_*D*_ 10 µM). At the highest Gal-3 concentrations, we have a direct binding between Gal-3 and anti-LAG-3-mAb (*K*_*D*_ 1 mM–1 µM) (Fig. 4F, G). Together, this confirms Gal-3-binding ability to not only LAG-3, but also directly to the antagonistic LAG-3 mAb and with the capacity to interfere with the Ag-mAb binding.

### Addition of Galectin-3 inhibitor to cell co-cultures did not change the cytokine production in neither PBMC nor SFMC cultures

The binding between Gal-3 and both LAG-3 and the LAG-3 mAb makes it possible that the biological effects of rhLAG-3 or antagonistic LAG-3 mAb are affected by Gal-3. Therefore, we tested the functional effect of adding a Gal-3 inhibitor to the PBMC and SFMC co-cultures with either rhLAG-3 or antagonistic LAG-3 mAb and measured cytokine production. As opposed to the expected, addition of the Gal-3 inhibitor resulted in no changes in the production of any of the cytokines measured (Fig. S5A, B).

Gal-3 is known to have a proinflammatory capacity, and adding the Gal-3 inhibitor alone also lowered the cytokine production. In this setup, adding rhLAG-3 gave rise to the same anti-inflammatory effect as the inhibition of Gal-3. The anti-inflammatory effect of Gal-3 was neutralized when blocking LAG-3 meaning that the Gal-3 effect at least partially works through the LAG-3 pathway.

## Discussion

LAG-3 is known to play an important immunoregulatory role as a checkpoint inhibitory receptor (CIR) in cancer, but the function of LAG-3 in autoimmunity, and RA in particular, is only sparsely described. Here, we show that levels of sLAG-3 are elevated in eRA, especially in patients with radiographic progression and the presence of autoantibodies. Furthermore, we show that levels of both soluble and membrane-bound LAG-3 are particularly high in the inflamed joint, and LAG-3 identifies a subpopulation of cells that is also PD-1 positive and produces IL-2 in chronic RA. Finally, we show that adding recombinant human LAG-3 results in decreased production of proinflammatory cytokines. This was not affected by inhibition of the LAG-3 ligand Gal-3. These findings support a hitherto unrecognized role of LAG-3 in dampening the inflammatory activity in RA.

In early RA, high baseline levels of sLAG-3 were primarily seen in IgM-RF or anti-CCP-positive patients and in patients with progression of bone erosions. This may seem counterintuitive due to the anti-inflammatory role of LAG-3. Different explanations may be offered to this finding. First, although the biological function of sLAG-3 still needs to be elucidated, sLAG-3 could act as a competing binding site to the ligands of the membrane-bound LAG-3. Second, as both ADAM10 and ADAM17 are highly expressed in RA [35, 36], cleavage of LAG-3 may drive the T cells to a less protective stage [8, 37]. Third, it might reflect an excessive immune reaction with sLAG-3 as an activation marker with a relative lack of LAG-3 inhibition resulting in a vain attempt to reduce the inflammatory burden. Finally, by binding to MHC-II, LAG-3 selectively inhibits the activation of T cells responsive to stable peptide-MHC-II complexes [10, 38]. Hence, the elevated sLAG-3 level may reflect the interference with antigen presentation and autoantibody production. The first 2 explanations support that sLAG-3 has direct pathogenic properties. Further research is needed to elucidate the mechanism of action of the abovementioned finding.

Although proteolytic cleavage and LAG-3 shedding by metalloproteases ADAM10 is increased 12-fold and ADAM17 is induced following T cell activation and TCR signaling [8], this does not affect the surface expression in RA significantly according to our results. RA patients in our study predominantly express LAG-3 on activated CD45R0 + memory-prone T cells primarily in the inflamed joint. They also co-expressed PD-1 and still had the ability to produce IL-2. Soluble LAG-3 is also 5–10 times higher in the synovial compartment. With the joint being the major site of inflammation, the elevated expression of LAG-3 in the synovial fluid, both the soluble and the membrane-bound, reflects an attempt to diminish this excessive immune reaction. Consistent with this, it has been reported that sLAG-3 continues to increase with continued activation, and the cleaved LAG-3 is replaced with newly synthesized LAG-3 [39]. In the plasma, high amounts of sLAG-3 reflect high disease activity (eRA, baseline) and continued T cell activation despite clinical remission (eRA, 12 months). In cRA, plasma levels above that of healthy controls, reflect the fact that cRA patients in this study are enrolled at a time of a flare and therefore inflammatory active.

Since most T cell-driven animal systemic arthritis models are highly similar to a type IV hypersensitivity reaction without the formation of anti-CCP antibodies and IgM-RF, we turned to an ex vivo model to further investigate the function of LAG-3 in RA. The ex vivo cultured RA synovial cell model is a unique model, representing actual diseased cells taken from the site of pathology, and is dominated by memory-prone T cells. It is driven by the spontaneous endogenous production of a diversity of pro-inflammatory factors and the production of autoantibodies. Using this setup, we showed that SFMCs from RA patients preserved their ability to produce large amounts of proinflammatory cytokines when activated through the CD3/CD28 pathway. We demonstrated that LAG-3 bioactivity plays a role in RA, as the addition of rhLAG-3 leads to a decrease in proinflammatory cytokines including TNFα and IFNγ, known to be pivotal for the pathogenesis in RA.

By its close similarity to CD4, LAG-3 is known to interfere with stable pMHCII, a central player in the mechanism of antigen processing and presentation [8]. This could be a central feature in the interpretation of the results since the affection of the downstream signaling of antigen processing, presentation, and potentially bidirectional inhibition takes time. The ex vivo model does not allow for a longer time course of experiments, which otherwise could have magnified the significance level of the results. Likewise, the direct LAG-3 effects could seem to be lower due to the investigation of bulk PBMC/SFMC instead of different purified T cell subtypes.

Gal-3 was investigated due to its alternative binding possibilities and inhibitory properties described in other contexts [6, 22, 40]. We confirmed LAG-3 binding to Gal-3 and interference of Gal-3 with the LAG-3/anti-LAG-3 mAb binding by the SPR/Evil Fit analysis. These results underscore that the presence of Gal-3 in increasing concentrations changes the binding kinetics between LAG-3 and its antibody from a typical antibody binding towards a trimolecular complex formation with Gal-3. This indicates that the potential clinical benefit of an anti-LAG3 mAb may be interfered within the presence of Gal-3 in the systemic circulation or locally.

However, the functional outcome of these potential large molecules was unpredictable. Theoretically, one could imagine Gal-3 multimers to both intensify the LAG-3 inhibitory effects, e.g., by gathering more LAG-3 molecules in the immunological synapsis, or minimize LAG-3 effects by interrupting the LAG-3-pMHC-II binding. It could also be discussed whether the Gal-3 concentration found to affect the LAG-3/LAG-3 mAb binding is physiologically relevant, but the concentration of Gal-3 in the microenvironment remains to be elucidated. Due to this, and the ubiquitous presence and promiscuous behavior of Gal-3, we chose to neutralize the Gal-3 effect instead of adding more. In this context, we observed that the addition of a Gal-3 inhibitor did not change the cytokine production induced by rhLAG-3 or antagonistic LAG-3 mAb alone, supporting the theory that Gal-3 concentrations needed to interrupt LAG-3/LAG-3 mAb binding are too high to be physiologically relevant. Again, direct LAG-3 effects may be lower in this setup due to the investigation of bulk T cells instead of distinctive T cell subtypes.

## Conclusion

In summary, the data presented herein propose LAG-3 as a faceted inhibitory receptor in the pathogenesis of RA with several key points to highlight (1) the increased level of LAG-3, particularly in the inflamed joint; (2) the positive correlation to autoantibody seropositivity and radiographic progression; and (3) the biologically active role by decreasing inflammatory cytokine production. This supports LAG-3 as a marker for continued T cell activation and local inflammation and as an important regulator in RA where persistent CD4 + T cell activation, antigen recognition, and bone degradation are key features. However, further in vivo studies are necessary in order to investigate the LAG-3 mechanisms of action and role in these processes but our observations support the relevance of doing so in RA as a model of a T cell-driven and MHC II-linked disease. Finally, we confirmed LAG-3/Gal-3 binding and a new finding showed that the functional outcome of LAG-3 and treatment with a LAG-3 mAb was not affected by Gal-3 interference, which is important as LAG-3 agonism could be a potential target for future treatment in RA.

## Supplementary Information

**Additional file 1: Fig. S1.** Gating strategy. A. Gating strategy for CD4/LAG-3, CD45R0/ LAG-3 and CD19/ LAG-3 measurement. B. Gating from FMO LAG-3.

**Additional file 2: Fig. S2.** Correlations of sLAG-3. A. The linear correlation between sLAG-3 levels at baseline and at 12 months (*r* = 0.7, *p*<0.0001). B. Logistic regression receiver operating curve (ROC) depicting s-LAG3 performance between the non-erosive (golden standard) and the erosive phenotype. The curve shows an overall performance of 67%, supporting s-LAG3 association with the risk of developing erosions in eRA.

**Additional file 3: Fig. S3.** Cellular expression of LAG-3 in PBMCs and SFMCs. A. Cellular expression of LAG-3 on CD3+CD4- T cells from PBMCs and SFMCs in HC (*n *= 6) and cRA (*n* = 9) B. Distribution of LAG-3+ cells in relation to CD45R0 presented in the bar graph (*n* = 9).

**Additional file 4: Fig. S4.** Cellular expression of LAG-3 in CD19+ B cells. A. Cellular expression of LAG-3 on CD19+ B cells on PBMCs and SFMCs from chronic RA patients. B. A representative flow plot from one of the patients.

**Additional file 5: Fig. S5.** Effects of rhLAG-3 and anti-LAG3-mAb on cytokine production in the presence of a Gal-3 inhibitor. Production of cytokines in recombinant human LAG-3 treated (A) and anti-LAG3-mAb (B) treated PBMC and SFMC cultures from RA patients (*n* = 11) with or without addition of Galectin-3 inhibitor. Level of significance is indicated by * < 0.05. Differences were analyzed using the Mann-Whitney test to compare two groups.

## Data Availability

The data used and analyzed during the current study are available from the corresponding author upon reasonable request.

## Abbreviations

ACR: American College of Rheumatology
ADA: Adalimumab
ADAM10/17: A disintegrin and metalloproteinase domain-containing protein 10/17
Anti-CCP: Anti-cyclic citrullinated peptide
BE: Bone erosions
CD: Cluster of differentiation
CIRs: Co-inhibitor receptors
cRA: Chronic rheumatoid arthritis
CRP: C-reactive protein
CTLA-4: Cytotoxic T-lymphocyte-associated protein 4
DAS28CRP: Disease Activity Score 28 for Rheumatoid Arthritis with CRP
DMSO: Dimethyl sulfoxide
EDTA: Ethylen diamin tetra acetat
ELISA: Enzyme-linked immunosorbent assay
eRA: Early rheumatoid arthritis
Fc: Fragment crystallisable
FCS: Fetal calf serum
FGL-1: Fibrinogen-like protein 1
FMO: Fluorescence Minus One
Gal-3: Galectin 3
HC: Healthy controls
HLA II: Human leukocyte antigen cc
IFNγ: Interferon gamma
IgM-RF: Immunoglobulin M rheumatoid factor
IL: Interleukin
IQR: Interquartile range
JSN: Joint space narrowing
*K*_*a*_: Association constant
*K*_*D*_: Dissociation constant
*K*_off_: Rate constant for dissociation
LAG-3: Lymphocyte activation gene 3
LAG-3 mAb: Lymphocyte activation gene 3 monoclonal antibody
LSECtin: Liver and lymph node sinusoidal endothelial cell C-type lectin
MHC-II: Major histocompatibility complex class II
MTX: Methotrexate
NK cells: Natural killer cells
OD: Optical density
OPERA: OPtimized treatment in Early RA
PBMCs: Peripheral blood mononuclear cells
PD-1: Programmed cell death protein 1
pMHC-II: Peptide-MHC class II
RA: Rheumatoid arthritis
rh-Gal-3: Recombinant human Galectin-3
rh-LAG-3: Recombinant human lymphocyte activation gene 3
RPMI: Roswell Park Memorial Institute
SF: Synovial fluid
SFMCs: Synovial fluid mononuclear cells
sLAG-3: Soluble lymphocyte activation gene 3
SPR: Surface plasmon resonance
TCR: T cell receptor
TNFα: Tumor necrosis factor alpha
Tregs: Regulatory T cells
TSS: Total Sharp Score
V-PLEX: Validated multiplex assay
