# Supplementary figures and images for: Lymphocyte activation gene 3 is increased and affects cytokine production in rheumatoid arthritis
Source: Arthritis Res Ther. 2023 Jun 7;25:97. doi: 10.1186/s13075-023-03073-z (PMC10246404; doi:10.1186/s13075-023-03073-z)

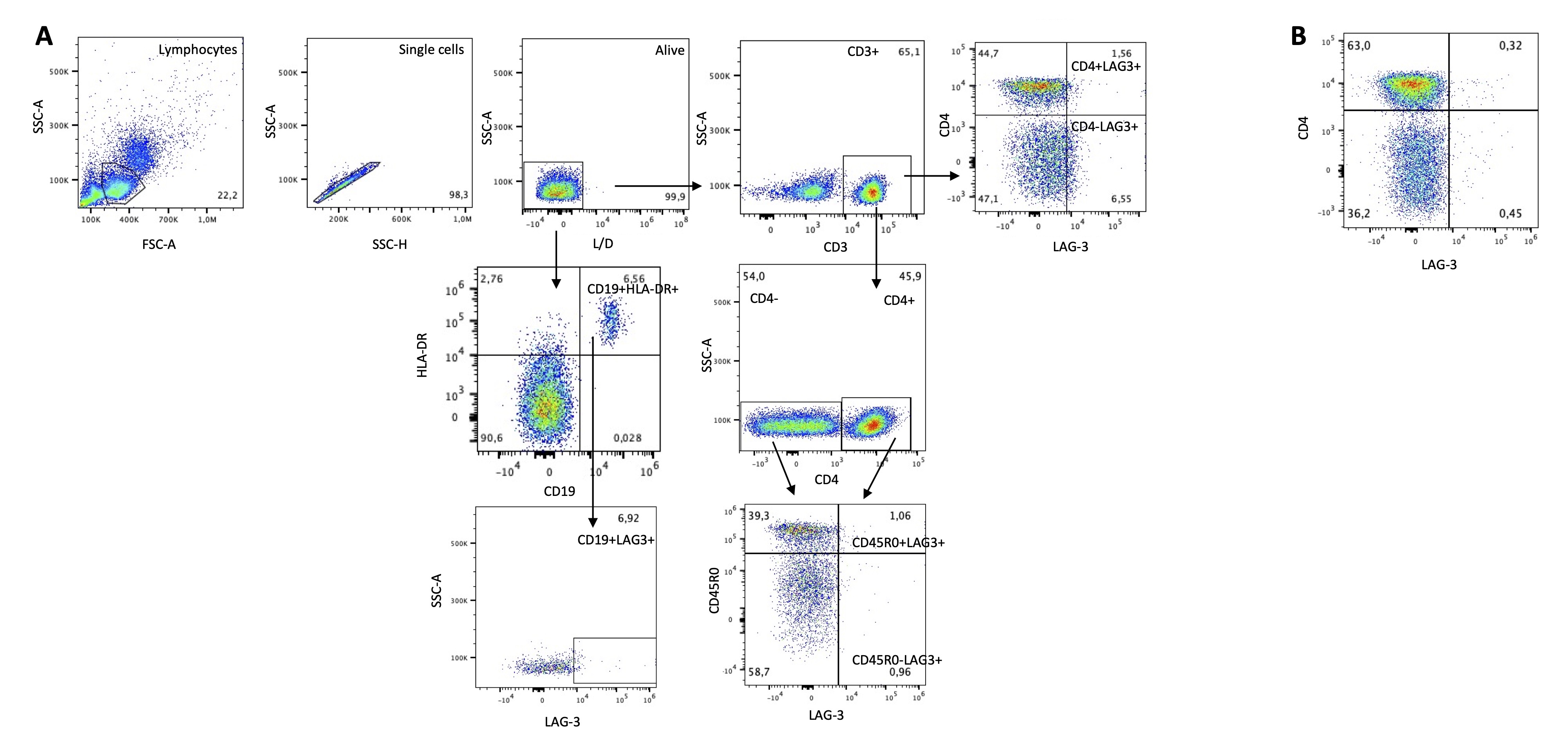

Supplement: Supplementary file 1 — Additional file 1: Fig. S1. Gating strategy. A. Gating strategy for CD4/LAG-3, CD45R0/ LAG-3 and CD19/ LAG-3 measurement. B. Gating from FMO LAG-3. [file 13075_2023_3073_MOESM1_ESM.jpg]

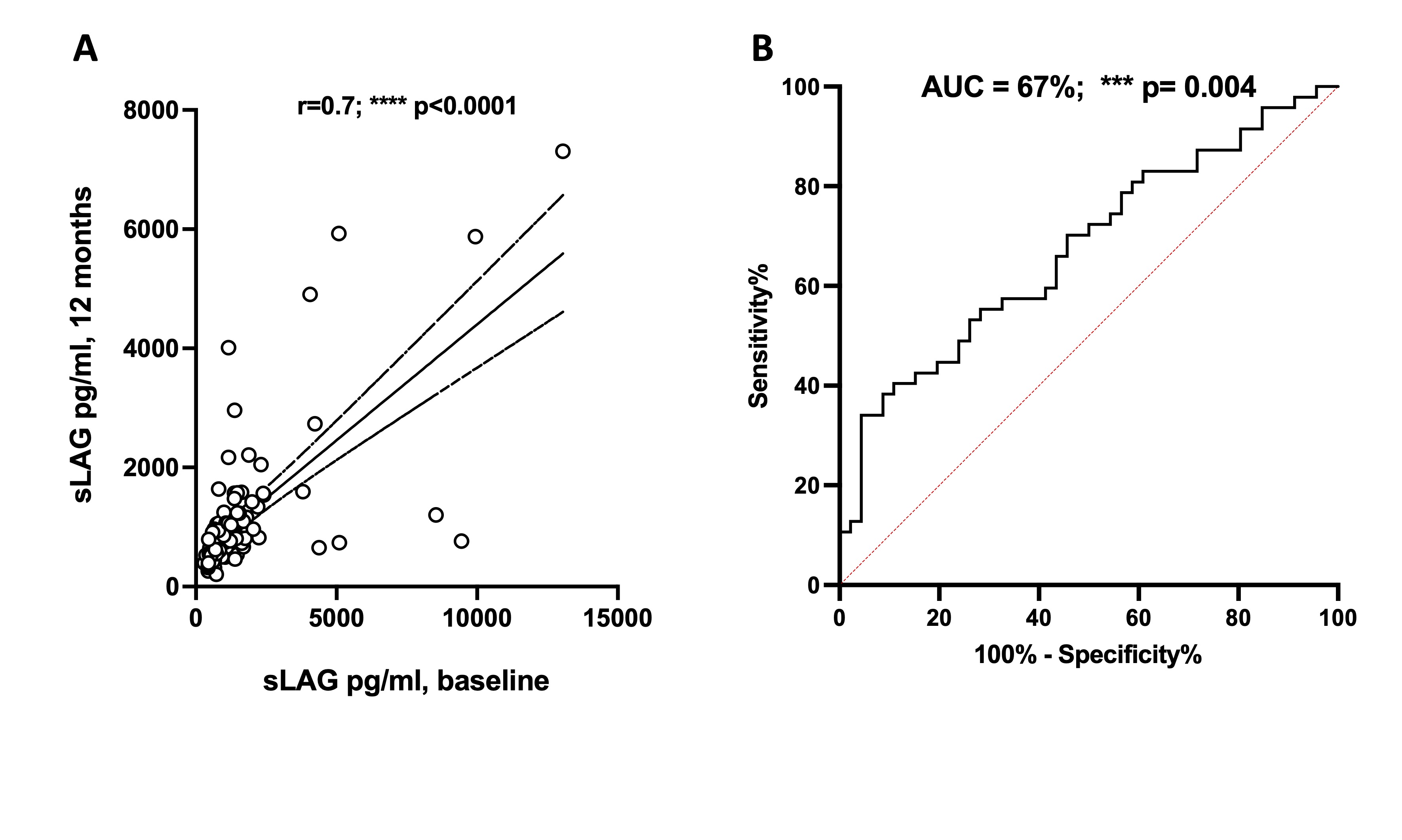

Supplement: Supplementary file 2 — Additional file 2: Fig. S2. Correlations of sLAG-3. A. The linear correlation between sLAG-3 levels at baseline and at 12 months (r = 0.7, p<0.0001). B. Logistic regression receiver operating curve (ROC) depicting s-LAG3 performance between the non-erosive (golden standard) and the erosive phenotype. The curve shows an overall performance of 67%, supporting s-LAG3 association with the risk of developing erosions in eRA. [file 13075_2023_3073_MOESM2_ESM.jpg]

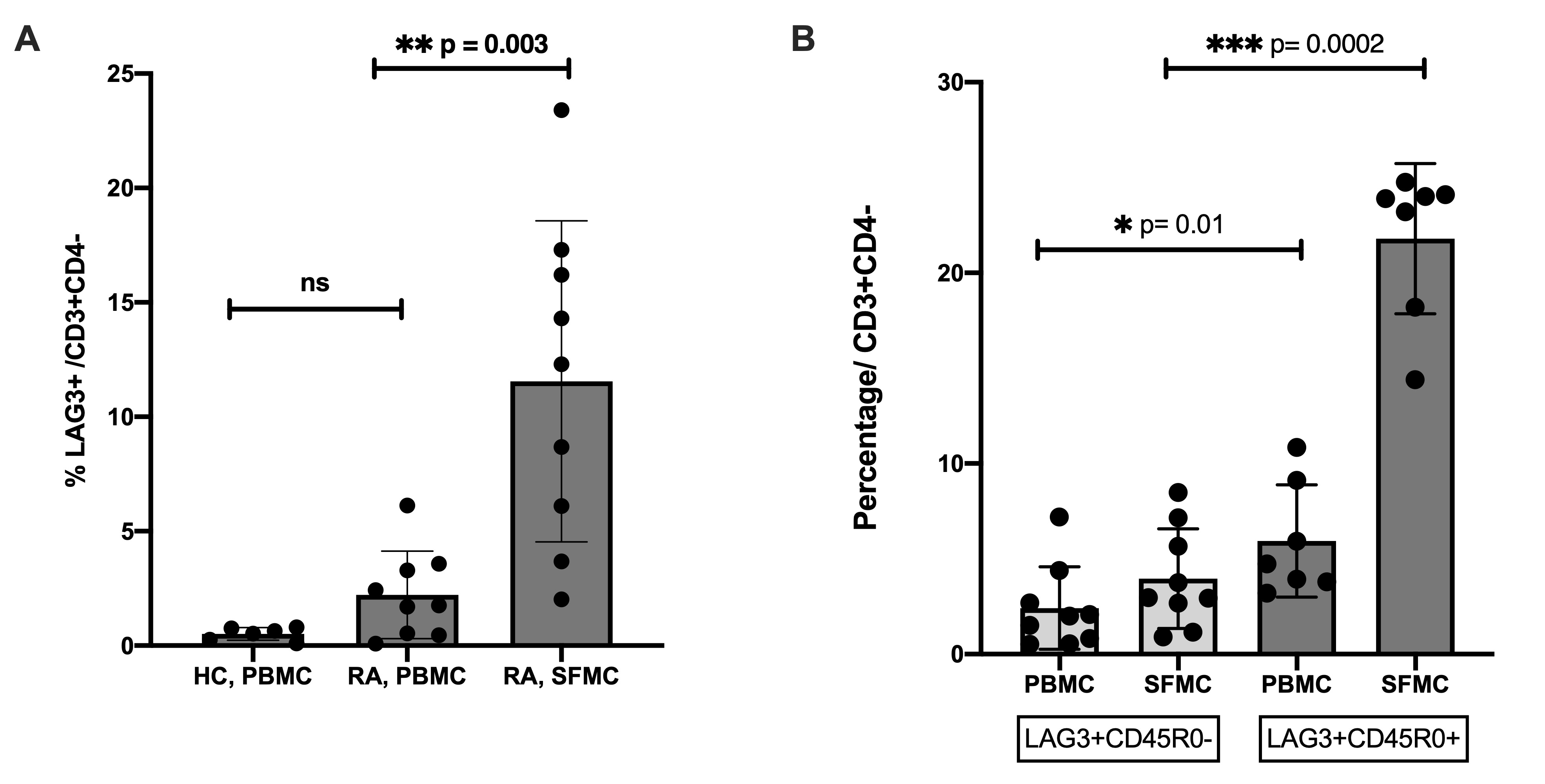

Supplement: Supplementary file 3 — Additional file 3: Fig. S3. Cellular expression of LAG-3 in PBMCs and SFMCs. A. Cellular expression of LAG-3 on CD3+CD4- T cells from PBMCs and SFMCs in HC (n = 6) and cRA (n = 9) B. Distribution of LAG-3+ cells in relation to CD45R0 presented in the bar graph (n = 9). [file 13075_2023_3073_MOESM3_ESM.jpg]

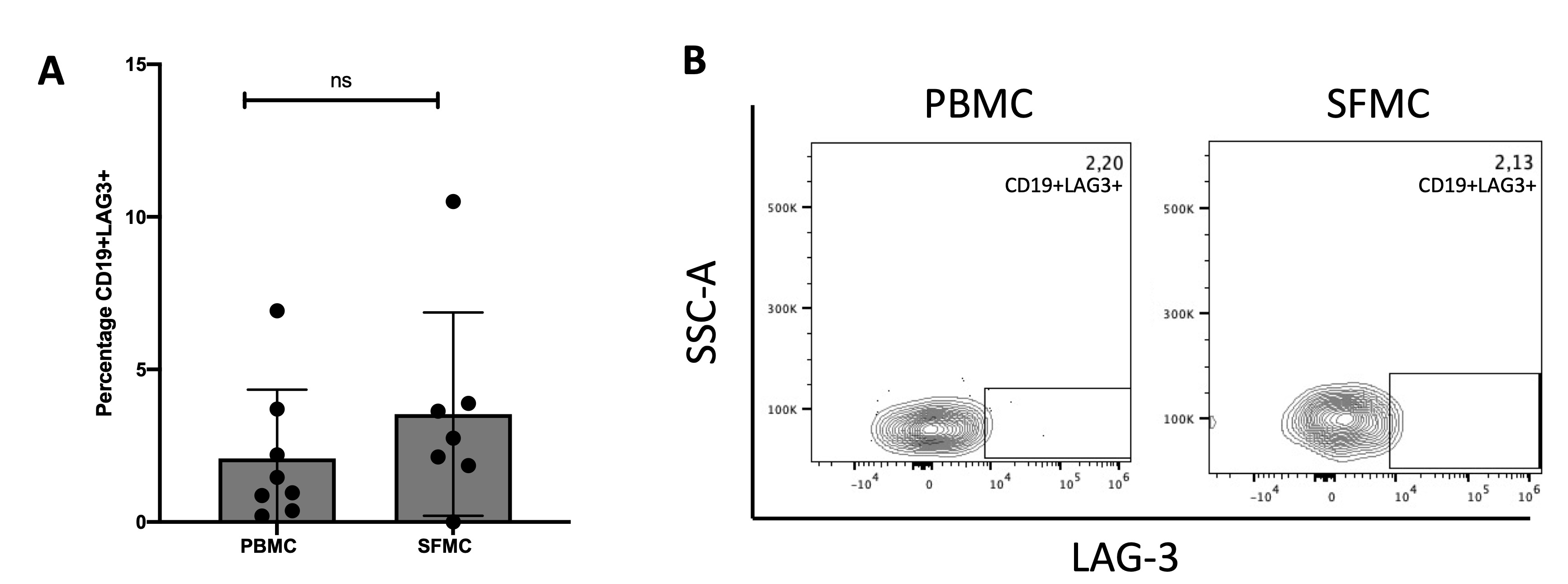

Supplement: Supplementary file 4 — Additional file 4: Fig. S4. Cellular expression of LAG-3 in CD19+ B cells. A. Cellular expression of LAG-3 on CD19+ B cells on PBMCs and SFMCs from chronic RA patients. B. A representative flow plot from one of the patients. [file 13075_2023_3073_MOESM4_ESM.jpg]
